# Electrified bioreactors: the next power‐up for biometallurgical wastewater treatment

**DOI:** 10.1111/1751-7915.13992

**Published:** 2021-12-19

**Authors:** Pieter Ostermeyer, Luiza Bonin, Luis Fernando Leon‐Fernandez, Xochitl Dominguez‐Benetton, Tom Hennebel, Korneel Rabaey

**Affiliations:** ^1^ Faculty of Bioscience Engineering Center of Microbial Ecology and Technology (CMET) Ghent University Coupure Links 653 Ghent B‐9000 Belgium; ^2^ CAPTURE Frieda Saeysstraat 1 Ghent 9000 Belgium; ^3^ Separation and Conversion Technology Flemish Institute for Technological Research (VITO) Boeretang 200 Mol 2400 Belgium; ^4^ Group Research and Development, Competence Area Recycling and Extraction Technologies Umicore Watertorenstraat 33 Olen B‐2250 Belgium

## Abstract

Over the past decades, biological treatment of metallurgical wastewaters has become commonplace. Passive systems require intensive land use due to their slow treatment rates, do not recover embedded resources and are poorly controllable. Active systems however require the addition of chemicals, increasing operational costs and possibly negatively affecting safety and the environment. Electrification of biological systems can reduce the use of chemicals, operational costs, surface footprint and environmental impact when compared to passive and active technologies whilst increasing the recovery of resources and the extraction of products. Electrification of low rate applications has resulted in the development of bioelectrochemical systems (BES), but electrification of high rate systems has been lagging behind due to the limited mass transfer, electron transfer and biomass density in BES. We postulate that for high rate applications, the electrification of bioreactors, for example, through the use of electrolyzers, may herald a new generation of electrified biological systems (EBS). In this review, we evaluate the latest trends in the field of biometallurgical and microbial‐electrochemical wastewater treatment and discuss the advantages and challenges of these existing treatment technologies. We advocate for future research to focus on the development of electrified bioreactors, exploring the boundaries and limitations of these systems, and their validity upon treating industrial wastewaters.

## Graphical abstract

Over the past decades, biological treatment of metallurgical wastewaters has become commonplace. Electrification of low rate biological applications has resulted in the development of bioelectrochemical systems (BES), but electrification of high rate systems has been lagging behind due to the limited mass transfer, electron transfer and biomass density in BES. We postulate that for high rate applications, the electrification of bioreactors, e.g., through the use of electrolyzers, may herald a new generation of electrified biological systems (EBS).

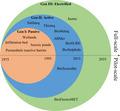

## Introduction

Over the past decades, the advent of renewables, electrification and decarbonization has led to a steep increase in the demand for metals, specifically the so‐called critical metals (United States of America, 2012; Zepf *et al*., 2014; Hennebel *et al*., 2015; European Commission, 2020). This has resulted in the intensification of mining and metallurgical operations, such as (bio)leaching, smelting, roasting, electrowinning and electrorefining, which, in turn, result in the production of toxic wastewater containing pollutants and otherwise prospectable resources (Northey *et al*., 2014). The wastewaters can be the byproduct of spontaneous processes such the biological oxidation of sulfide ores, resulting in the production of acid mine drainage, or the result of (bio)leaching, treatment of off‐gasses from smelters, roasting installations, or bleeds from electrowinning and refining tank houses (Johnson and Hallberg, 2005; Simate and Ndlovu, 2014; Rambabu *et al*., 2020). They are characterized by an acidic pH (< 4), high sulfate concentrations (ca. 5–15 gSO_4_^2−^ l^−1^), the absence of organics and various embedded metals and metalloids such as iron, copper, cadmium, arsenic, selenium, tellurium, aluminium, lead, nickel, zinc and manganese, with concentrations varying from 1 µg l^−1^ to 10 g l^−1^ (Hallberg and Johnson, 2005; Peiravi *et al*., 2017; Ostermeyer *et al*., 2020). Treatment is required as they constitute an environmental and safety hazard. Although pollutants such as arsenic or cadmium in the wastewater need to be removed and immobilized into stable forms, some of the metals, such as copper, selenium and tellurium, are valuable and should be recovered in pursuit of circularity. Sulfur compounds can also be recovered and water reused, allowing for maximal recovery of the embedded resources and minimal production of waste.

The occurrence of acid mine drainage has been known since antiquity, but the treatment of these wastewaters has only gained traction since the rise of the environmental movement in the 1970–1980s (Secundus, 79AD; Skousen *et al*., 2017). Technologies used for the treatment of acid, metal and sulfate‐containing waters have been developed and adapted to treat acid mine drainages at abandoned mines (Gazea *et al*., 1996; Johnson and Hallberg, 2005; Skousen *et al*., 2017). Treatment of metallurgical wastewaters based on the use of alkali (e.g. limestone, (un)hydrated lime) was initially dominant. In this type of treatment, compounds such as CaCO_3_ are used to neutralize the acidity of the wastewater, precipitate metals as hydroxides, remove sulfate as gypsum (CaSO_4_·2H_2_O) and arsenic as calcium arsenate (Ca_3_(AsO_4_)_2_). This approach suffers from limestone depletion, high use of chemical, production of high volumes of waste and high operational costs (Johnson and Hallberg, 2005; Gopi Kiran *et al*., 2017). Additionally, traditional metal hydroxide precipitation is unviable for removing metals and metalloids down to levels that adhere to increasingly stringent environmental standards (Lewis, 2010). Plus, the targeted metals are not selectively removed, and many end up diluted in the waste sludge, which is typically landfilled (Huisman *et al*., 2006; Riveros *et al*., 2014; Simate and Ndlovu, 2014).

**Fig. 1:**
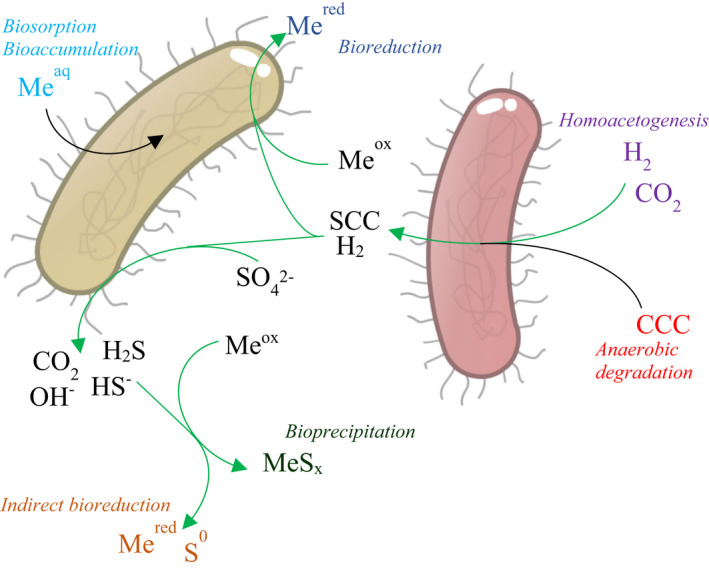
Syntrophic relationship and metal(loid) removal mechanisms at play in biological systems for the treatment of sulfate containing metallurgical wastewaters. CCC, complex carbon chains; SCC, short carbon chains. Green arrows denote pathways that can be electrified.

Biological technologies used for remediation of acid mine drainage and metallurgical wastewaters can counter these disadvantages (Gregory and Lovley, 2005; Johnson and Hallberg, 2005; Willquist *et al*., 2015). They are chiefly based on sulfate reduction: in the presence of an electron donor such as a biodegradable organic compound or hydrogen gas, sulfate‐reducing microorganisms such as *Desulfovibrio* spp. and *Desulfomonas* spp. will reduce sulfate through anaerobic respiration (Muyzer and Stams, 2008). This results in the partial neutralization of the acid and the bioprecipitation of metals as metal sulfides (Kaksonen and Puhakka, 2007). Other mechanisms such bioreduction, biosorption and bioaccumulation may also contribute to biological metal removal (Fig. 1) (Nancharaiah *et al*., 2015b).

During the reduction of sulfate, acidity is consumed as H^+^ and alkalinity is generated as HCO_3_^−^ (pKa 6.35) or HS^−^ (pKa 7.04). Within this environment, metals and metalloids coprecipitate as metal sulfides or can be reduced via (indirect) bioreduction. Since the initial use of sulfate‐reducing microorganisms for remediation of acid mine drainage in the 1970–1980s, the associated technology has evolved from passive treatment (“Generation I”) to active treatment (“Generation II”) (Kaksonen and Puhakka, 2007). In this review, we first relate the history of sulfate reduction based biotechnologies from this first generation of passive treatment to the second generation of active treatment. Finally, based on the challenges encountered by these past generations, trends observed in other biotechnological fields and the societal push towards increased electrification [e.g. bioelectrochemical systems (BES)], it is postulated that a third generation of electrified biological systems (EBS) is ready to propel high rate biometallurgical wastewater treatment into the age of electrification.

## Generation I: Passive biological treatment

Passive treatment is the oldest application of sulfate‐reducing microorganisms for remediation of acid mine drainage (Skousen *et al*., 2017). In passive treatment, an anaerobic system is constructed using organic matter and limestone or other alkalinity generating rock. The wastewater flows gravimetrically through the constructed treatment system without the use of additional energy. The limestone neutralizes the acidity and provides alkalinity. Sulfate‐reducing microorganisms use organic matter to reduce sulfate to sulfides. The embedded metals and metalloids subsequently precipitate as sulfides in the treatment system. Typical examples of such passive biological systems are permeable reactive barriers, infiltration beds, anoxic ponds, anoxic drains and constructed (aerobic or anaerobic) wetlands (Johnson and Hallberg, 2005; Kaksonen and Puhakka, 2007; Skousen *et al*., 2017).

Microorganisms utilized in these systems must be resilient towards fluctuations in pH, flow rate and metal concentration, as the treatment system is poorly controllable and subjected to variable conditions (Kaksonen and Puhakka, 2007; Neculita *et al*., 2007). These parameters may vary depending on mining activities, groundwater levels, level of storage ponds and precipitation in the area. Factors such as temperature, depth, flow direction, water height and the presence of nitrate or (micro)aerobic zones, affect the diversity, viability and succession of the microbial community and may select for aerotolerant sulfate reducers, denitrifiers and sulfide oxidizers such as *Thiobacillus* spp., possibly hampering the neutralizing capacity of the treatment (Pruden *et al*., 2006). Depending on the (an)aerobic character of the environment and the elements embedded in the wastewater, the growth of microorganisms metabolizing iron, selenium, arsenic and manganese can be promoted (Hallberg and Johnson, 2005; Lindsay *et al*., 2011; Parissa Mirjafari and Susan, 2012; Baldwin *et al*., 2015). When a complex organic source such as sewage, compost or molasses is used, sulfate‐reducing microorganisms must be supplemented with *ad hoc* organic matter degrading microorganisms, such as *Fibrobacter* spp., *Pseudoxanthomonas* spp. and *Brevundimonas* spp., that break down the complex organic matter to molecules that can be used by the sulfate‐reducing microorganisms through anaerobic digestion/fermentative pathways (Hallberg *et al*.; Liamleam and Annachhatre, 2007; Skousen *et al*., 2017). The variability in operational parameters and the complexity of the organic matter may also select for organisms such as *Desulfobacteria* spp., which are more versatile in terms of substrate use than, for example, *Desulfovibrio* spp. (Pruden *et al*., 2006).

These systems were quickly implemented as they are a low‐cost method for remediation of acid mine drainage and removal of pollutants, requiring a minimum of labor, chemicals and maintenance and no utilities. Disadvantages of these systems are their poor design, controllability and predictability, the risk of clogging, formation of preferential flow channels, the consumption of organic matter and limestone, the unselective precipitation of metals, the in‐situ precipitation of large volumes of waste, the low achieved rates (see a non‐exhaustive summary of reported rates in Fig. 4), the resulting high footprints and effluents that might not meet environmental standards and regulations (Johnson and Hallberg, 2005; Kaksonen and Puhakka, 2007).

## Generation II: Active biological treatment

Passive systems (“Generation I”) have been designed to treat acid mine drainage and metallurgical wastewaters with relatively low flow rates in remote locations, where sufficient area was available to accommodate the large footprint of the treatment systems. These systems are less suited to treat wastewater from metallurgical plants such as urban smelters. These wastewaters are characterized by high flow rates, concentrated acidity (pH < 4), high concentrations of sulfate (ca. 15 gSO_4_^2−^ l^−1^), metals and pollutants (e.g. 2 g As l^−1^), which require high rate treatment technologies (Kaksonen and Puhakka, 2007).

**Fig. 2:**
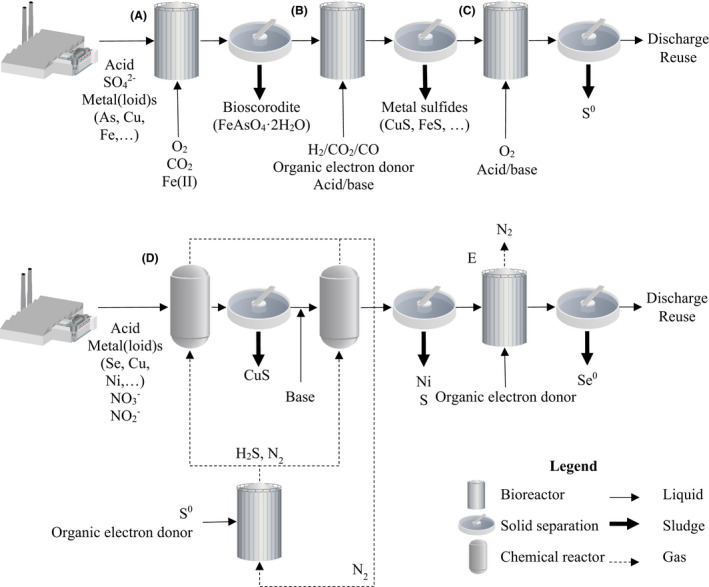
Overview of possible hypothetical flowsheets using multiple biometallurgical wastewater treatment technologies. (A) Arsenoteq/Thioteq Scorodite, (B) Sulfateq, (C) Thiopaq, (D) Thioteq or BioSulphide with selective recovery, (E) BioMeteq or ABMet. These conceptual flowsheets are non‐limiting as multiple iterations, sequences, combination, gas recycles and liquid recycles are possible.

Active sulfate and sulfur‐reducing bioreactors were developed as a second generation of treatment systems for concentrated wastewaters and high flows. Various reactor types have been developed and used as sulfate‐reducing bioreactors, such as continuously stirred tank reactors, fluidized bed reactors, membrane bioreactor and gas lift reactors, and have been commercialized as Sulfateq™ and Thioteq™ by Paques, BioSulphide™ by BQE water and the BioSURE™ process (see Table 1) (van Houten, 2006; Papirio *et al*., 2013; Rose, 2013; Isosaari and Sillanpää, 2017). Treatment systems have been operated in various configurations using a combination of gas or liquid recycles, offline bioreactors using externally added S^0^ and multiple stages of bioreactors, chemical reactors, settlers and strippers. The multitude of configurations in which these bioreactors can be implemented in metal processing flowsheets allows for the flexible design of treatment systems tuned to the specific challenges and composition of the targeted waste stream (see Fig. 2) (Kaksonen and Puhakka, 2007). The embedded metals and metalloids can be targeted for selective recovery, and the embedded sulfur can be recovered when the treatment is combined with (biological) oxidation of sulfides to elemental sulfur (Thiopaq™) or stripping and conversion of H_2_S to SO_2_, which can be valorized as sulfuric acid (Van Lier *et al*., 1999). Full‐scale examples of such flowsheets can be found at the Grootvlei Gold Mine (South Africa), the Budel plant of Nyrstar (The Netherlands), the Kennecott mine (Utah, USA), the former Britannia mine (Canada) and the Pueblo Viejo goldmine (Dominican Republic) (Boonstra *et al*., 1999; Hulshoff Pol *et al*., 2001; Kaksonen *et al*., 2020). Besides bioreactors developed with the express purpose of reducing sulfates, the BioMeteq™ and ABMet™ technologies have been developed for the remediation or recovery of nitrate, nitrite, selenium, chromium and uranium and also for the precipitation of other metals as metal sulfides through reduction of relatively small quantities of sulfates (Weijma *et al*., 2007; GE Power and Water, 2013; Zhuang *et al*., 2015). The most recent bioreactor technology developed for the treatment of metallurgical arsenic‐bearing wastewater is the Arsenoteq/Thioteq™ Scorodite technology, an aerobic process wherein As(III) and Fe(II) are biologically oxidized and coprecipitated in atmospheric conditions as highly stable bioscorodite (FeAsO_4_·2H_2_O) (González‐Contreras *et al*., 2012).

**Table 1:** Overview of developed biometallurgical wastewater treatment technologies.

Technology	Electron donor	Electron acceptor	Organisms	Example species (non‐limiting)	Goal
BioSulphide (Isosaari and Sillanpää, 2017) Thioteq (Huisman *et al*., 2006)	Organic (e.g. ethanol and molasses)	S^0^	Sulfate reducers Fermenters	*Desulfovibrio* spp. *Desulfomonas* spp. *Fibrobacter* spp *Desulphuromonas acetoxidans*	Generate H_2_S for metal(loid) sulfide removal/recovery
BioSURE (Rose, 2013)	Sewage sludge	SO_4_^2−^	Sulfate reducers Fermenters	*Desulfovibrio* spp. *Desulfomonas* spp.	Metal(loid) removal/recovery
Sulfateq (van Houten *et al*., 1995)	H_2_, CO, Organic (e.g. ethanol, molasses)	SO_4_^2−^	Sulfate reducers Fermenters Acetogens	*Desulfovibrio* spp. *Desulfomonas spp*. *Acetobacterium* spp. *Fibrobacter* spp.	Sulfate removal Metal(loid) sulfide removal/recovery
ABMet (GE Power and Water, 2013) BioMeteq (Weijma *et al*., 2007)	Organic (e.g. ethanol and molasses)	NO_3_^−^ NO_2_^−^ SO_4_^2−^ Metal(loid)s (e.g. Se, As, U and Cr)	Sulfate reducers Fermenters Metal(loid) reducers Denitrifiers	*Desulfovibrio* spp. *Desulfomonas* spp. *Fibrobacter* spp. *Bacillus arsenoselenatis* *Thauera selenatis* *Sulfurospirillum barnesii*	Denitrification Metal(loid) removal/recovery
Thiopaq (Ter Heijne *et al*., 2018)	HS^−^, H_2_S	O_2_	Sulfide oxidizers	*Thioalkalivibrio, Sulfolobulus* *Alkalilimnicola* spp.	S° recovery
Arsenoteq/Thioteq Scorodite (Gonzalez‐Contreras *et al*., 2010)	Fe(II) As(III)	O_2_	Iron oxidizers Arsenite oxidizers	*Acidianus sulfidivorans, Thiobacillus ferrooxidans* *Thermus* spp. *Sulfolobus acidocaldarius*	As removal

Various electron donors are used in sulfate‐reducing bioreactors, whose respective advantages and disadvantages have been discussed in other reviews (Larry *et al*., 1995; Kaksonen and Puhakka, 2007; Liamleam and Annachhatre, 2007; Papirio *et al*., 2013). The employed electron donor affects the microbial community of the systems. When H_2_ is used as an electron donor and CO_2_ is used as a carbon source, either an autotrophic sulfate‐reducing community or a syntrophic culture of autotrophic acetogens and heterotrophic sulfate reducers develops (Van Houten *et al*., 2009). CO can also be used as carbon source and electron donor (e.g. in syngas), but care must be taken due to the toxic nature of CO (Parshina *et al*., 2010). When short‐chain electron donors are used, such as acetate, the community may be limited to heterotrophic sulfate reducers. When more complex substrates such as molasses are used, a syntrophic community is required. The complex substrates first need to be broken down by organic matter degrading microorganisms to short carbohydrates, which can be subsequently used by sulfate reducers as electron donor (Fig. 1) (Liamleam and Annachhatre, 2007). Depending on the concentration of electron donor and carbonates present, methanogenesis may occur, resulting in loss of electron donor and methane gas emission (Van Houten *et al*., 2006). Technologies designed for removal of metal(loid)s and nitrate/nitrite utilize denitrifying and metal(loid)‐reducing organisms such as *Bacillus arsenoselenatis, Thauera selenatis* and *Sulfurospirillum barnesii* (Nancharaiah and Lens, 2015). When these treatment systems are combined with other biological treatment technologies used for elemental sulfur or bioscorodite precipitation, iron (*Acidianus sulfidivorans* and *Thiobacillus ferrooxidans)*, arsenic (*Thermus* spp., *Sulfolobus acidocaldarius*) and sulfide (*Thioalkalivibrio, Sulfolobulus* and *Alkalilimnicola* spp.) oxidizing organisms are used (Van Lier *et al*., 1999; Gonzalez‐Contreras *et al*., 2010; de Rink *et al*., 2020). The pH, temperature and concentrations of metals, organic acids and H_2_S influence the community and efficacy of the system (Hulshoff Pol *et al*., 2001; Kaksonen *et al*., 2004; Kaksonen and Puhakka, 2007; Sánchez‐Andrea *et al*., 2014).

In general, these treatment systems are characterized by relatively high sulfate reduction rates (see Fig. 4), increased resource recovery, improved process control and relatively low footprints. The Sulfateq installation built by Paques and Nyrstar at Budel, for example, reaches sulfate reduction rates of 14.2–19.2 gSO_4_^2−^ l^−1^ d^−1^. In literature, reduction rates up to 29 gSO_4_^2−^ l^−1^ d^−1^ have been reported (van Houten *et al*., 1994, 2009). Whilst the reduction rate and related reactor size determine the capital expenditure (CAPEX), the operational cost (OPEX) is determined by the type and amount of electron donor, and carbon source used (Bijmans, 2008; Sleutels *et al*., 2012). Additionally, alkali has to be added to the bioreactor to maintain a stable circumneutral pH, especially when acidic wastewater containing high sulfate levels is treated (Van Houten *et al*., 2006; Bijmans *et al*., 2008). The purchase of these chemicals does not only increase the OPEX of the operating systems but also implies the off‐site production, transport and storage of the chemicals. This often results in increased costs, environmental impact and the burdens related to safety and environmental regulations associated with the transport and storage of the chemicals. Finally, the use of these chemicals may result in residual organic matter in the discharged water or may result in increased salinity, which can be problematic when the water is discharged into freshwater environments or is intended for onsite reuse (Liamleam and Annachhatre, 2007).

## Generation III: Electrified biological treatment

To avoid the use of chemicals and increasing the overall sustainability of treatments, several types of electrified bioreactors have been developed (Nancharaiah *et al*., 2015b; Dominguez‐Benetton *et al*., 2018). In these systems, electrical energy and electrochemical cells are generally used to replace the addition or regeneration of an external electron donor/acceptor or the addition of an alkali or acid for pH control (De Paepe *et al*., 2020; Folens *et al*., 2021). Additionally, products or resources can be extracted, purified or recovered (Verbeeck *et al*., 2018; Carvajal‐Arroyo *et al*., 2020). This new, third generation of electrified biological treatment can be broadly categorized in two broad groups: BES and EBS. In BES, microorganisms directly or indirectly exchange electrons with an electrode or electroactive surface, whilst EBS combine abiotic electrochemical cells with bioreactors (Wang and Ren, 2014; Nancharaiah *et al*., 2015a; Verbeeck *et al*., 2018).

### Bioelectrochemical systems (BES): childstars with growing pains

In BES, solid‐state electrodes serve as electron acceptors (bioanodes) or electron donors (biocathodes) to electroactive biological entities, such as microorganisms. When a BES yields net positive energy, it is classified as a microbial fuel cell (MFC), whilst in the case of microbial electrolysis cell (MEC) power is consumed to achieve reactions that are otherwise thermodynamically not feasible or slow. BES can be constructed via various combinations of cathodes, anodes, biocathodes and bioanodes. Several other reviews have discussed different configurations of BES, and their fundamental aspects are not the focus of the current review (Nancharaiah *et al*., 2015b; Dominguez‐Benetton *et al*., 2018). A non‐exhaustive summary of the BES configurations relevant to metallurgical wastewater treatment is given below.

BES research initially focussed on the development of bioanodes, where the biocatalyzed oxidation of organic compounds is used to generate power or drive the target reactions with relatively high redox potential on the cathode. In this configuration, some researchers have focussed on the development of bioanodes to drive the electrochemical reduction/deposition of dissolved metals in the abiotic catholyte. These works reported the removal of Cu, Ni, Cd and Zn (Ter Heijne *et al*., 2010; Qin *et al*., 2012; Colantonio and Kim, 2016; Modin *et al*., 2017). Modin *et al*. (2012) attained the selective sequential deposition of Cu, Pb, Cd and Zn from a simulated municipal solid waste incineration ash leachate by working under different cathode potentials in a MEC configuration (Modin *et al*., 2012). Luo *et al*. (2014b) achieved the simultaneous recovery of Cu, Ni and Fe from an artificial acid mine drainage (Luo *et al*., 2014b). Ai *et al*. (2020) achieved the selective recovery of Cu, Cd and Fe by working under MFC or MEC mode, and Leon‐Fernandez *et al*. (2021) the sequential recovery of Cu (in MFC mode) and Sn, Ni and Fe (in MEC mode), also from simulated acid mine drainages (Ai *et al*., 2020; Leon‐Fernandez *et al*., 2021). Finally, other studies have reported the coupling of bioanodes with the cathodic metal removal by precipitation as metal hydroxides or carbonates (Lefebvre *et al*., 2012; Colantonio and Kim, 2016). Likewise, electrochemical cells that can eventually be amended with microbial bioanodes have also achieved the selective precipitation of metal (hydr)oxides, mixed metal oxides or hydroxychlorides, using gas diffusion electrocrystallization (GDEx) (Prato *et al*., 2019, 2020; Pozo *et al*., 2020). GDEx can be adapted in both MFC and MEC modes, as necessary. In this way, a broad variety of metals can be recovered and valorized as functional or marketable materials. Sulfides too can be oxidized and recovered as elemental sulfur on a bioanode, whilst Fe(II) and As(III) can be biologically oxidized to Fe(III) and As(V), respectively (Rabaey *et al*., 2006; Ter Heijne *et al*., 2007; Nguyen *et al*., 2016).

The initial focus on bioanodes has pushed research into bioanodic based technologies (Bonmatí *et al*., 2013; Prévoteau *et al*., 2020). However, research and development of biocathodes has been lagging behind. This is peculiar, as metal(loid)s such as selenium, tellurium, arsenic, antimony, uranium, technetium, neptuntium, vanadium, silver, gold, cobalt, chromium, mercury, palladium and platinum can be immobilized, reduced in toxicity or transformed into valuable nanoparticles via bioreduction (Lloyd, 2003; Hennebel *et al*., 2012; Abin and Hollibaugh, 2014; Williamson *et al*., 2014; Maes *et al*., 2016). At the same time, sulfate reduction has been extensively used for the treatment of metallurgical wastewaters and removal or recovery of metal(loid)s in generation I and II systems (Johnson and Hallberg, 2005; Kaksonen and Puhakka, 2007). Biocathodes have been used for the reduction of toxic hexavalent chromium (Cr(VI)) to trivalent chromium (Cr(III)), the reduction of U(VI) to U(IV) and the biomediated cathodic reduction of Se(IV) and Se(VI) to zero‐valent state Se nanoparticles (Gregory and Lovley, 2005; Wang and Ren, 2014; Zhang *et al*., 2018; Jugnia *et al*., 2020). Cadmium, gold, vanadium, copper and cobalt have also been reduced using biocathodes (Mosquera *et al*.; Huang *et al*., 2014, 2015; Hao *et al*., 2015).

Biocathodic reduction of sulfate has been shown by multiple authors (see Table 2) (Su *et al*., 2012; Coma *et al*., 2013; Luo *et al*., 2014a; Sharma *et al*., 2014, 2015; Pozo *et al*., 2015, 2016, 2017a,b; Blàzquez *et al*., 2016; Blázquez *et al*., 2016, 2017; Teng *et al*., 2016; Agostino and Rosenbaum, 2018). Pozo *et al*. (2017b) was able to treat acid mine drainage in a chemical‐free way by using a biocathode combined with electrochemical cells, anion exchange membranes, cation exchange membranes and settlers (Pozo *et al*., 2017b). Sulfate was reduced to sulfides on the biocathode, sulfides were oxidized to elemental sulfur on an abiotic anode, and hydrogen evolution at an abiotic cathode was used to increase the pH of the acid mine drainage and precipitate metals as hydroxides (Pozo *et al*., 2017b). Blázquez *et al*. (2016) used an autotrophic biocathode for sulfate reduction whilst consuming the oxygen produced at the anode to oxidize the produced sulfides to elemental sulfur.

**Table 2:** Summary of sulfate‐reducing bioelectrochemical systems and a selection of other BES as a comparison.

Technology	Compound	Rate (g l^−1^ d^−1^)	Rate (g m^−2^ d^−1^)	Reference
Biocathode	SO_4_^2−^	0.02	1.8	Su *et al*. (2012)
Biocathode Bioanode	SO_4_^2−^	0.07	34	Coma *et al*. (2013)
Biocathode Bioanode	SO_4_^2−^	0.04	1.92	Luo *et al*. (2014a)
Biocathode	SO_4_^2−^	0.9	11	Pozo *et al*. (2015)
Biocathode Zn removal	SO_4_^2−^	0.04	0.35	Teng *et al*. (2016)
Biocathode In‐situ sulfide oxidation	SO_4_^2−^	1.16	16	Blàzquez *et al*. (2016)
Biocathode	SO_4_^2−^	1.5	86	Pozo *et al*. (2016)
Biocathode In‐situ sulfide oxidation	SO_4_^2−^	2.1	29	Blázquez *et al*. (2017)
Biocathode	SO_4_^2−^	16.8	330	Pozo *et al*. (2017)
Biocathode Abiotic electrodes	SO_4_^2−^	2.8	567	Pozo *et al*. (2017)
Inotec (pilot scale)	NO_3_^−^	0.5	n.d.	Opara *et al*. (2018)
BioElectroMET (labscale bioanode)	CH_3_COO^−^	n.d.	154	Rodenas Motos *et al*. (2015)
BioElectroMET (pilot‐scale bioanode)	CH_3_COO^−^	4.5	8	Rodenas Motos *et al*. (2017)
Fluidized bioanode	CH_3_COO^−^	0.005	9	Deeke *et al*. (2015)
Fluidized bioanode	Organic matter (O_2_)	1.7	723	Tejedor‐Sanz *et al*. (2018)
Fluidized biocathode	NO_3_^−^	0.137	58	Tejedor Sanz *et al*. (2020)

The use of BES for metallurgical wastewater treatment has remained mostly restricted to the lab scale. Two rare examples of the pilot‐ and full‐scale application of BES are the BioElectroMET and Inotec technologies (Adams *et al*., 2012; Rodenas Motos *et al*., 2017). The BioElectroMET technology was developed by Rodenas Motos *et al*. (2015) and is based on the use of a MEC in which the bioanodic oxidation of acetate drives the abiotic electrodeposition of copper on the cathode, with associated production of electricity. By scaling up the aforementioned bioreactors with an electrode area of 100 cm², maximum current densities of 23 A m^−2^ were achieved (Rodenas Motos *et al*., 2015). However, when the scale was increased from an electrode surface area of 100 cm^2^ to 700–835 cm^2^, current densities dropped to 1.2 A m^−^², which was attributed to increased internal resistances of the cell, lower anode coulombic efficiencies due to the degradation of acetate through non‐bioelectrochemical pathways and due to poor cell design and electrochemical engineering (Rodenas Motos *et al*., 2017). The Inotec technology is based on a BES for the treatment of wastewater polluted with selenium, nitrate, nitrite, uranium, sulfate and other metal(loid)s. A cathode and anode are inserted into packed bed bioreactors with activated carbon as a biocarrier, wherein external voltage ranging from 1 to 3 V is applied. Nitrates and nitrites are reduced to N_2_ gas and metal(loid)s precipitate in the bed through bioreduction or bioprecipitation as metal(loid) sulfide. This technology allows reducing OPEX and CAPEX associated costs by 25–50% and has been used at pilot and full scale. At pilot scale, a flow rate of 180 l h^−1^ was used, and concentrations up to 114 mg NO_3_^−^ l^−1^ and 2.7 mg Se l^−1^ have been successfully treated (Adams *et al*., 2012; Opara *et al*., 2018). It is however impossible to exclude the occurrence of non‐electroactive microbial nitrate, sulfate or metal(loid) reduction as the technology still employs some organic carbon source. Furthermore, no provisions have been made for the recovery of the precipitates from the bed, which would require backwashing, flushing or replacement of the biocarrier.

The advantages of BES‐based technologies are clear: minimization of chemicals and OPEX, the possibility of power generation in the case of MFCs, increased availability of electron donor (i.e. H_2_) and reduction of employed voltages due to the bioelectrocatalytic effect of the electroactive microorganisms (Pozo *et al*., 2017a; Cecconet *et al*., 2018; Dominguez‐Benetton *et al*., 2018). An added advantage is the possibility of achieving a selective and sequential recovery of the target metals, and eventually an easier production of marketable and functional materials (Logan *et al*., 2008; Sleutels *et al*., 2012). However, the implementation of BES remains challenging: In addition to the characteristics listed in generation I, the employed microorganisms often must be electroactive (Arends *et al*., 2014; Ntagia *et al*., 2016; Izadi *et al*., 2019). Secondly, the electrodes have to be biologically compatible, display low electrical resistance and provide a large surface area for electron exchange and biofilm growth (Desloover *et al*.; Guo *et al*., 2015; Sharma *et al*., 2019). Most importantly, when scaling up, it has proven to be difficult to attain sufficiently high rates (see Table 2) (Sleutels *et al*., 2012; Prévoteau *et al*., 2020). The reported sulfate reduction rates vary from 0.002 to 2.8 gSO_4_^2−^ l^−1^ d^−1^ (see Table 3 and Fig. 4) (Coma *et al*., 2013; Pozo *et al*., 2017b). The Inotec technology has been applied on a full scale to treat acid mine drainage, brines from membrane treatment and power station wastewater. A rate of 0.5 g NO_3_^− ^l^−1^ d^−1^ was reported, which would equate to 0.4 gSO_4_^2−^ l^−1^ d^−1^ if sulfate was the relevant electron acceptor (Opara *et al*., 2018). Achieved reduction rates per area of electrode are also too low to warrant scale up, varying from 0.35 to 567 gSO_4_^2−^ m^−^² d^−1^, negatively affecting reactor size and CAPEX (Kaksonen and Puhakka, 2007; Rozendal *et al*., 2008; Su *et al*., 2012; Pozo *et al*., 2017a). For bioanodic MEC CAPEX is estimated at 100 € m^−^² (Sleutels *et al*., 2012). As biocathodes currently achieve current densities up to 10 times lower than bioanodes, the CAPEX of biocathodic systems can be estimated to be 10 times higher (Sharma *et al*., 2014).

**Table 3:** Advantages and disadvantages of passive, active and electrified biological systems for the treatment of metallurgical wastewater (Johnson and Hallberg, 2005; Taylor *et al*., 2005; Kaksonen and Puhakka, 2007; Skousen *et al*., 2017; Verbeeck *et al*., 2018).

	Chemical based		Electrified	
	Gen I: Passive treatment	Gen II: Active treatment	Gen IIIa: BES	Gen IIIb: Electrified bioreactors
Resource recovery	Difficult	Yes	Possible^a^	Yes
Footprint (m²)^b^	Very high	Low	High	Low
Chemical use	Low/medium	High	Low	Low
OPEX	Low	High	Low	Low
Rate	Low	High	Low	High
CAPEX	Medium	Determined by reduction rate	High	Medium

^a^ Resource recovery in the bioelectrochemical systems (BES) is dependent on the implemented configuration: resource recovery from wetland‐like systems is difficult, whil*st* recovery from reactor‐like systems is feasible.

^b^ Assuming a volume‐surface area of 3.8 m³ m^−2^ for reactors, a flow rate of 40 m³ h^−1^ and a sulfate concentration of 15 gSO_4_^2−^ l^−1^: Very high: 14 000–4 300 000 m^2^; High: 1300–190 000 m^2^; Low: 130–350 m^2^.

One possible route for scale up of BES is maximizing the bioelectroactive surface area. Fluidized bioanodes and biocathodes employing capacitive biocarriers have been successfully used, as shown by Tejedor‐Sanz *et al*. (2020, 2018) (Deeke *et al*., 2015; Tejedor‐Sanz *et al*., 2018; Tejedor Sanz *et al*., 2020). These fluidized BES still require additional research and have not yet achieved the rates of the active sulfate‐reducing bioreactors of generation II, which are required to treat high flows of concentrated metallurgical wastewater (see Table 3 and Fig. 4).

### Electrified biological systems (EBS): uncoupling as the fastest route to scale up

As the electron and mass transfer between the electrode and microorganisms and the amount of microorganisms able to grow on the electroactive surface remain the limiting factor for achieving sufficiently high rates, a different route for scaling up can be the decoupling of electrodes and biofilms. Hydrogenotrophic active sulfate‐reducing bioreactors utilized in generation II have been able to attain the reduction rates required to treat concentrated metallurgical wastewater at high flow rates (see Fig. 4). Commercial electrolyzers operate at 10 000–30 000 A m^−2^, resulting in electrodes 200–1 000 000 times smaller than those used in BES (Proost, 2019; Lee *et al*., 2020). Instead of transferring the electrons directly to and from microorganisms, an intermediary product such as hydrogen gas, products of CO_2_ reduction (e.g. as CO and formic acid), iron and oxygen gas can be generated at the electrode (Ter Heijne *et al*., 2007; Izadi *et al*., 2019). As abiotic electrochemical cells can achieve higher current densities than BES, coupling these cells to high rate bioreactors similar to those developed for generation II may unlock the high required rates that have proven elusive for BES, provided gas transfer is managed adequately (Sleutels *et al*., 2012).

In the context of metals, electrochemistry and electrometallurgy are widely used for electrorefining and electrowinning in concentrated electrolytes (Verbruggen *et al*., 2020). Recent research has shown the potential of electrochemical cells to reduce chemical use and extract embedded compounds from dilute streams. Electrochemical cells have been used for the recovery of copper, cobalt, zinc, ammonia and terephthalic acid from various dilute metal‐containing streams, for the in‐situ production of acid, the precipitation of PbSO_4_ from lead citrate leachate and leaching and extraction of rare earth elements (Maes *et al*., 2017; Gao *et al*., 2020, 2021; Folens *et al*., 2021). Alternatively, GDEx has been used for the recovery of Fe, Cu, Zn, Co, Mn, As and the platinum group metals (Prato *et al*., 2019, 2020; Pozo *et al*., 2020). In the PLATIRUS project, platinum group metals have been selectively recovered (70–100%) out of complex metallurgical mixtures with > 40 different and more concentrated metals (Nicol *et al*., 2021).

**Fig. 3:**
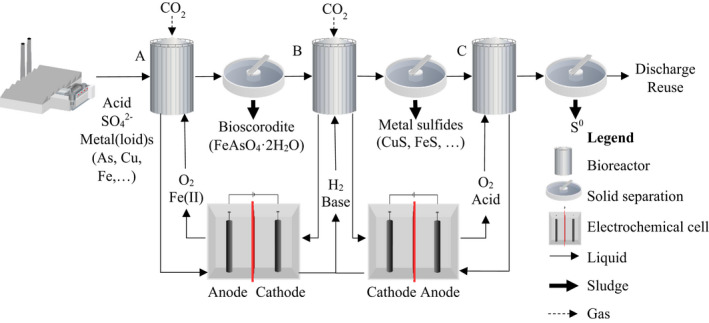
A hypothetical example of electrified biological systems (EBS) applied to existing biometallurgical wastewater treatment technologies. (A) Arsenoteq/Thioteq Scorodite, (B) Sulfateq and (C) Thiopaq. This conceptual flowsheets is non‐limiting as multiple iterations, sequences, combination, gas recycles and liquid recycles are possible.

The coupling of such electrochemical systems to a bioreactor used for the treatment of metallurgical wastewater has not been studied, but other research areas already have combined electrochemical cells and bioreactors into electrified bioreactors. Electrified bioreactors similar to the system employed in Verbeeck *et al*. (2018) and De Paepe *et al*. (2020) (see Table 4) may be able to treat metallurgical wastewater, coupling the in‐situ production of hydrogen gas, base and acid in the electrochemical cell with the reduction of sulfate and metalloids, the precipitation of metal(loid)s and the general extraction of resources (Verbeeck *et al*., 2018; De Paepe *et al*., 2020). Not only reductive systems could be coupled to electrochemical cells. Aerobic and oxidative treatments can also be electrified, such as biological oxidation of sulfides and precipitation as elemental sulfur or the precipitation of bioscorodite (Gonzalez‐Contreras *et al*., 2010; Vaiopoulou *et al*., 2016; Ntagia *et al*., 2019). Biological sulfide oxidation requires the supply of oxygen and an acid or base to stabilize the pH (Ter Heijne *et al*., 2018; de Rink *et al*., 2019). The precipitation of bioscorodite involves the supply of oxygen and Fe(II), which can be provided via the in‐situ oxidation of iron at the anode (see Fig. 3) (Gonzalez‐Contreras *et al*., 2010).

**Table 4:** Non‐exhaustive overview of the characteristics, performance and application of electrochemical systems and electrified biological systems.

System	Goal (X)	Membrane	Electrodes	Current density (A m^−2^)	Cell potential (V)	Rate (gX l^−1^ d^−1^)^a^	Reference
ES	Extraction of Nd and La	CEM, AEM	A: MMO‐Ir Ti C: Stainless steel	40	9–13	–	Maes *et al*. (2017)
	Water electrolysis	PEM	A: Ni coated steel C: Ni coated steel	10 000–30 000	1.5–3	–	Lee *et al*. (2020)
	Co recovery	AEM	A: MMO‐Ir Ti C: Stainless steel	50	7–28	–	Gao *et al*. (2020)
	Pb and citrate recovery	CEM	A: MMO‐Ir Ti C: Stainless steel	50–100	3	–	Folens *et al*. (2021)
	Cu and Zn extraction	AEM	A: MMO‐Ir Ti C: Stainless steel	93	3	–	Gao *et al*. (2021)
	Cu recovery (Cu)	None	A: Ti mesh C: Copper	Up to 700	2	Up to 8	Haccuria *et al*. (2017)
BES	Cu recovery	Bipolar membrane	A: Rough graphite plate C: Copper	4.5	−0.5^b^	0.17	Ter Heijne *et al*. (2010)
	Cu, Ni and Fe recovery	Bipolar membrane	A and C: Graphite brush	4.4	1	0.003	Luo *et al*. (2014a)
	Cd(II) removal	None	A: Carbon fiber brush C: Stainless steel mesh	1.9	1	–	Colantonio and Kim (2016)
EBS	Acetate production and extraction (CH_3_COO^−^)	CEM, AEM	A: MMO‐Ir Ti C: Carbon felt	5	3.91	1.48–3.54	Verbeeck *et al*. (2018)
	Nitrification (NH_4_^+^‐N)	CEM, AEM	A: MMO‐Ir Ti C: Stainless steel	20	2.8–3.5	0.24	De Paepe *et al*. (2020)
	Cr precipitation (Cr)	CEM	A and C: stainless‐steel mesh	9	2.4	0.01	Anaya‐Garzon *et al*.

A, anode; AEM, anion exchange membrane; BES, bioelectrochemical systems; C, cathode; CEM, cation exchange membrane; EBS, electrified biological systems; ES, electrochemical systems; MMO, mixed metal oxide.

^a^ “X” refers to compound listed in the second column (“Goal”).

^b^ Negative voltage implies spontaneous voltage input (microbial fuel cell mode).

As described previously, the chemical consumption by sulfate‐reducing bioreactors determines the OPEX of the system and results in multiple downsides (Van Houten *et al*., 2006; Bijmans *et al*., 2008; Bijmans, 2008). Electrochemical cells can remediate these issues via the in‐situ production of acids, alkalis, electron donor, electron acceptor and the extraction/recovery of embedded compounds (Verbeeck *et al*., 2018; De Paepe *et al*., 2020). Although the required applied cell voltage will possibly be higher than in biocatalyzed electrochemical systems, these electrochemical cells will be less constrained by the low electron transfer rate, small area available for the growth of microorganisms and mass transfer limitations within the biofilm (Prévoteau *et al*., 2020). The combination of electrochemical cells and bioreactors such as in Fig. 3 can minimize the OPEX of the operated systems. This is dependent on the local electricity price, the applied voltage, the amount of current required and the coulombic efficiency of the system, all of which require further in‐depth research. Furthermore, the installation of electrolyzers and electrochemical cells will increase the CAPEX when compared to the treatment systems of generation II. Compared to classical BES, however, the limited electrode area and bioreactor volume required in these electrified bioreactors may result in lower CAPEX. Additionally, when the treatment is fully electrified, the possibility for carbon neutral metallurgical wastewater treatment opens up if the electricity is generated by renewable sources. Finally, these electrified bioreactors could provide a solution to remote mines and metallurgical plants, where the transport of chemicals may result in substantial additional costs and environmental burdens (Kaksonen and Puhakka, 2007).

## A place for everything, everything in its place

The low rates achieved by BES do not mean there is no room for BES applications (Bejan and Bunce, 2015). Low rate passive biological treatments such as anaerobic wetlands, permeable reactive barriers, anoxic ponds and infiltration beds have been widely utilized for the treatment of acid mine drainage. These systems can be amended with electrodes to improve their treatment efficiency, chemical use and longevity. The Inotec system previously described consists of electrodes inserted into an activated carbon bed. This system can be interpreted as a passive infiltration bed amended with electrodes, which results in a reduction of chemical use and operational costs (Opara *et al*.; Peoples and Adams, 2010). This working principle has also been shown by Prado *et al*. (2020) by improving wetlands as “METlands^®^”, which results in enhanced organic matter and nitrogen removal, increased removal rates and a reduced footprint. The use of an electroactive and conductive biocarrier allows the minimization of the electrode surface area and the associated CAPEX (Prado *et al*., 2020).

The advantages and key characteristics of passive treatment, active treatment, BES and electrified bioreactors are listed in Table 3. From this table, it is clear that passive biological treatment is suitable for handling low rate waste streams in remote areas such as acid mine drainage. In these areas, sufficient space is available to accommodate the achieved low rates. Due to the low value and remoteness of the waste stream, the treatment system must be low in maintenance and chemical consumption (Gazea *et al*., 1996; Kaksonen and Puhakka, 2007). By electrifying these passive biological treatment systems into BES similar to those used by Prado *et al*. (2020), the footprint and chemical use of the treatment can be further reduced, decreasing the operational costs and environmental impact (Opara *et al*.; Peoples and Adams, 2010; Prado *et al*., 2020). Compared to passive biological treatment, active biological treatment provides several advantages, for example, high treatment rates of concentrated metallurgical wastewater, increased operational control, a reduction of the footprint and higher resource recovery (Kaksonen and Puhakka, 2007). The OPEX of these systems is determined by their chemical consumption (Van Houten *et al*., 2006; Bijmans *et al*., 2008; Bijmans, 2008). Future research should aim to combine the low OPEX of BES with the advantages of active biological treatment, resulting in a new generation of high rate electrified bioreactors suitable for the treatment of high flows of concentrated metallurgical wastewater.

## The road to follow: electrified high rates

**Fig. 4:**
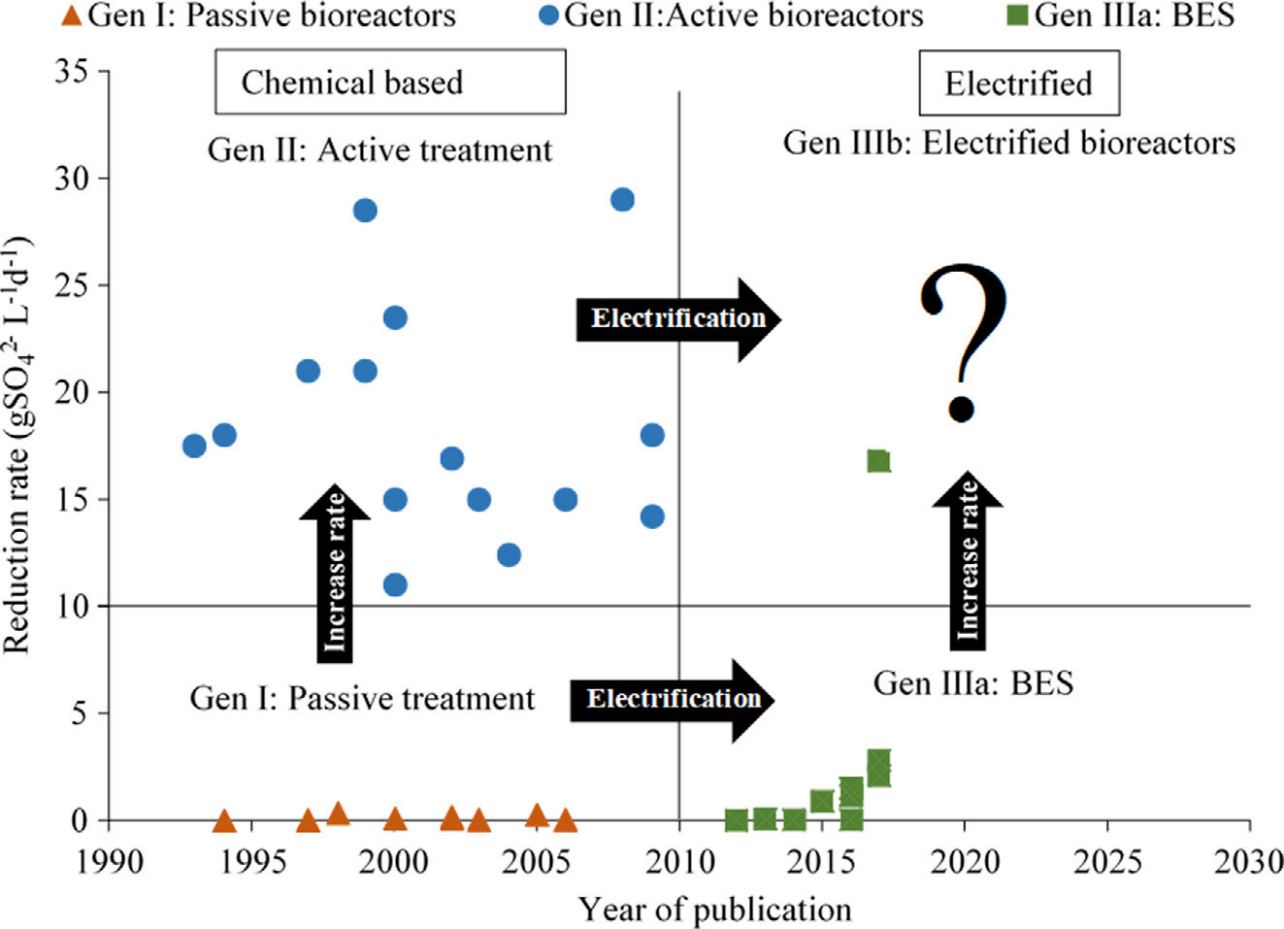
Reduction rates achieved by passive treatment (generation I), active treatment (generation II) and bioelectrochemical systems (BES, generation IIIa) reported in peer‐reviewed journals and the innovations between each generation (Dvorak *et al*., 1992; Smul *et al*., 1997; Waybrant *et al*., 1998, 2002; Chang *et al*., 2000; Cocos *et al*., 2002; Skousen and Ziemkiewicz, 2005; Zagury *et al*., 2006; Liamleam and Annachhatre, 2007; Van Houten *et al*., 2009; Su *et al*., 2012; Coma *et al*., 2013; Luo *et al*., 2014a; Sánchez‐Andrea *et al*., 2014; Sharma *et al*., 2014; Pozo *et al*., 2015, 2016, 2017, 2017; Blázquez *et al*., 2016, 2017; Teng *et al*., 2016).

Historically, two trends can be observed (Fig. 4). The first one concerns the maximization of sulfate reduction rate and the development of high rate active biological systems (generation II) to treat concentrated (pH < 4, ca. 15 gSO4^2−^ l^−1^) wastewaters at high flows, which are unsuitable for passive treatment (generation I). This has resulted in multiple mature full‐scale technologies used to treat high rate metallurgical wastewater, reporting sulfate reduction rates above 10 gSO_4_^2−^ l^−1^ d^−1^ (van Houten, 2006; Papirio *et al*., 2013; Rose, 2013; Isosaari and Sillanpää, 2017). The second trend consisted of the development of BES and electrification of low rate systems such as filtration beds and wetlands (Adams *et al*., 2012; Prado *et al*., 2020). This resulted in a minimization of operational costs and a decrease in footprint.

These BES remain for the foreseeable future unsuitable for the treatment of wastewater from metallurgical plants such as smelters, which are characterized by high flows, high concentrations and often very low pH (Kaksonen and Puhakka, 2007; Prévoteau *et al*., 2020). The biological treatment of these types of wastewaters has remained the exclusive domain of active biological treatment technologies (van Houten, 2006; Papirio *et al*., 2013; Rose, 2013; Isosaari and Sillanpää, 2017). These technologies still require the use of chemicals such as electron donors, acids and alkalis. These chemicals increase the OPEX of the treatment, increase the salinity of the wastewater and lower the potential for reuse (Van Houten *et al*., 2006; Bijmans *et al*., 2008; Bijmans, 2008). The off‐site production, transport and storage of these chemicals also result in additional costs, administrative burdens, safety hazards and environmental impacts, certainly when the plant is located in a remote location (Kaksonen and Puhakka, 2007).

Table 4 provides a comparison of some examples of electrochemical, bioelectrochemical and EBS. High current densities are a limiting factor on the electroactive biofilm performance of BES. BES allow to achieve a more favourable energetic balance compared to conventional electrochemical technology thanks to biocatalysis, abating energy requirements (Wang and Ren, 2014). However, current densities exceeding the biological capacity of the electroactive biofilm lead to biofilm inhibition and damage, limiting the reaction rates and deteriorating performance (Modin *et al*., 2012). Pure electrochemical reactors can operate at current densities some orders of magnitude higher than electrified bioreactors. This can be seen when comparing BES for copper removal (Ter Heijne *et al*., 2010) with pure electrochemical technology (Ter Heijne *et al*., 2010; Haccuria *et al*., 2017; Haccuria *et al*., 2017). Electrode materials for biofilm development need to be biocompatible. Carbonaceous porous materials are commonly used, which allow a proper development of the electroactive biofilm. In contrast to BES, the bioprocesses in EBS are uncoupled from the electrochemical reaction. Current densities and materials are therefore not restricted by biological limitations and could have a wide range depending on the particular case. BES and EBS need to operate at mild pH and temperature values to maintain the microbial community.

The achieved current density in some of the cited electrochemical systems and electrified bioreactors is however still (much) lower than in commercial electrolyzers. In part, this can be explained by the non‐optimal circumstances in labscale setups: a large distance between electrodes, for example, increases the cell potential significantly. Such factors can be remediated when knowhow of full‐scale operational electrolyzers is implemented. The low/mild conductivity of biological media, which also functions as an electrolyte, presents a fundamental challenge however. High conductivities are required to minimize the cell potential and energy consumption. One promising strategy to minimize the cell potential is the use of brines in the electrolysis cells. These brines can originate from onsite side or wastestreams or can be produced from the treated wastewater when the electrified bioreactor is combined with membrane treatment such as reverse osmosis. Crucially, this approach also requires the use of halotolerant and halophilic microorganisms: current research has shown that sulfate reducing bioreactors can operate at conductivities of 60–90 mS cm^−1^ (Vallero *et al*., 2004). Finally, the employed membranes and electrodes must be able to withstand the electrolyte pH and corrosive substances such as sulfides.

Given the long history of electrometallurgy and electrochemistry in metallurgical industries, the use of abiotic electrochemical cells for the electrification of active biological treatment technologies is a natural next step (Davy, 1839). This approach has the potential to combine the high treatment rates and low footprints achieved by active bioreactors with the advantages of BES. Future research should therefore focus on providing proof of concepts of these electrified bioreactors, focussing on the factors that determine the OPEX and CAPEX of the system: the chemical use, the total energy used, the employed potential and current, the achieved coulombic efficiency, the overall cell resistance, the water/electrolyte/medium conductivity and the achievable rates (Kaksonen and Puhakka, 2007; Prévoteau *et al*., 2020). Researchers should also keep the potential for resource recovery and water reuse in mind and investigate any existing boundaries limiting real‐world application. Examples of these may be the lack or presence of certain compounds and undesirable side reactions. Arsenic, for example, can be electrochemically reduced to arsine gas, a highly toxic and potentially lethal gas (Brusciotti and Duby, 2007). Other unwanted reactions such as the precipitation of hydroxides may result in the clogging of membranes. Metal(loid)s such as mercury, copper and silver can be reduced and/or deposited on the cathode, whilst elements such as chloride can be oxidized at the anode. Compounds such as fluoride or chlorine may deteriorate membranes, result in corrosion and damage electrodes and electrode coating. Finally, these systems must be tested and validated via the actual industrial wastewater treatment to increase the acceptance in industry and bridge the “valley of death” often encountered by nascent technologies (Hennebel *et al*., 2015).

## Conflict of interest

The authors declare no competing financial interest.
